# From ‘Immoral’ Users to ‘Sunbed Addicts’: The Media–Medical Pathologising of Working-class Consumers and Young Women in Late Twentieth-century England

**DOI:** 10.1093/shm/hkac012

**Published:** 2022-04-29

**Authors:** Fabiola Creed

**Keywords:** sunbeds, skin cancer, moral panic, addiction, mass media

## Abstract

Drawing on the changing representations of sunbed consumers within everyday entertainment media and national newspapers from the late 1980s to early 1990s, this article will demonstrate how sunbed use was framed, at first, as an ‘immoral’ working-class activity, and later as a growing addictive threat to white adolescent women. Medical experts had finally confirmed that sunbeds increased the risk of developing skin cancer, and the media had taken this ‘public health’ matter into their own hands. As this occurred during a backlash against Thatcherism, their anti-sunbed coverage became entangled with moralised concerns about class, women and consumerism. These sunbed warnings stigmatised both ‘yuppies’ and young women who exercised their new economic freedoms. Unravelling these complex political, economic and social tensions will also show how historians can use fictional and ‘low-brow’ media sources (from television soaps, cartoons and the *Daily Mail*) to further develop the history of public health approaches.

In 1978, sunbeds were first introduced in the UK. For years, people in Britain perceived the use of these expensive sunbeds as a luxurious, moral and rational activity for those who were affluent, ‘beautiful’ and both health- and fitness-conscious. But over time ‘cowboy salons’ and shops began to dominate the sunbed market. Working-class manufacturers had started producing cheap sunbeds in bulk, making the units accessible for the working-class masses. As sunbeds were now consumed by more people (and were now framed as a banal technology), dermatologists and medical physicists then conducted experiments to assess if sunbeds caused skin cancer.^1^ During the late 1980s, these scientists used these studies to somewhat confirm that sunbed use did increase the risk of developing skin cancer. It was only ‘somewhat confirmed’ because they wanted to conduct long-term studies. Nonetheless, this confirmation, from ‘authoritative’ voices and backed up by ‘science’ published in long-established and credible medical journals, helped justify the media's undesirable stereotyping of sunbed users—even if this was unintended by medical experts. As such, the media’s ‘public health’ messages against sunbed use were delivered with confidence and remained unchallenged.^2^

The main purpose of this article is to show that the media who produced everyday popular culture took health matters into their own hands. The first section of this article will show how the media first created, and then reinforced a fictional ‘immoral’ sunbed stereotype during the late 1980s. This stereotype was depicted through the national print press (both the *Guardian* and the *Daily Mail*), a radio show, a television soap, a *Punch* cartoon and later through films. This wide-range coverage suggests greater audience perception, and both radio shows and television soaps were more interactive mediums than just newspapers. These influential broadcasts would have reached far beyond the print press’s segmented audiences, instead reaching nation-wide audiences. Yet this entertainment media remains underused by medical historians.

By evaluating print press coverage of genuine sunbed users, the second section demonstrates how these ‘immoral’ sunbed users morphed into real-life ‘tanorexics’ or ‘sunbed addicts’ during the early 1990s. The *Daily Mail* led the ongoing association of ‘tanorexia’ with white adolescent women, which dermatologists and psychologists did not challenge, and instead endorsed.^3^ In media–medical coverage, the real risk of sunbed-induced skin cancer became entangled with the moralised concerns about class, women and consumerism. Even to this day, the representation of young, white and working-class ‘tanorexics’, as self-destructive, narcissistic and ‘stupid’, still persists in British ‘sunbed addiction’ documentaries.^4^

As such, this article seeks to historicise how and why working-class consumers and white adolescent women, rather than any other demographic groups, were pathologised for using sunbeds during the late 1980s and 1990s. This will illuminate wider economic, political, medical and socio-cultural changes and tensions within late twentieth-century Britain, which were reflected within these media-transmitted ‘health’ messages; thus, building on the history of working-class stigma, moral panic, gendered addiction and the contradictory expectations of women’s bodies and social roles.

To historicise ‘tanorexia’, the term first appeared colloquially in American newspapers during the 1980s.^5^ In 1991, it appeared in the British media for the first time, tellingly, through the *Daily Mail*.^6^ In this *Daily Mail* article, Dr Prem Misra (a senior consultant psychiatrist working for the Greater Glasgow Health Authority), claimed that he was the first medical authority to formally coin the term in Britain. He described ‘tanorexia’ as a ‘psychological addiction to sunbathing—either on a sunbed or in the sun’. Misra claimed that sunbed addiction ‘affected young women, and some young men’. From then onwards, both medical experts and the media defined ‘tanorexia’ as an obsessive desire to acquire and maintain a permanent deep tan by using tanning machines. Individuals with this ‘psychological disorder’ perceived themselves as pale, regardless of how darkened their skin became.^7^ During the early 1990s, sunbed-related concerns then peaked in medical journals.^8^ In these journals (and later books), the participants selected for these sunbed studies consisted of either entirely or mostly white adolescent women, often in full-time employment and/or educated.^9^ By the twenty-first century, ‘tanning addiction’ was understood by medical and public health authorities as both a psychological and physical addiction to the ultraviolet (UV) radiation of sunbeds.^10^ Sunbed ‘addiction’ was analogised to tobacco and alcohol addiction, as the ‘addict’ was said to experience severe withdrawal symptoms if they reduced or terminated their tanning consumption.^11^ Yet, ‘Tanorexia’ provides a unique case study as sunbed use had abruptly shifted from being unmistakably healthy to unmistakably dangerous.

To fully appreciate the political and medical agendas at play in the creation of ‘tanorexia’, it is important to critically assess the strength of the contested link between sunbeds and skin cancer from the outset. Malignant melanoma remains one of the most aggressive forms of cancer, and dermatologists claim that it is the most common skin cancer caused by sunbeds.^12^ Since the 1950s, mortality rates have doubled every 10–12 years, leading to a current death rate of approximately 1,500 people a year in Britain.^13^ The validity of the link between sunbeds and melanoma mortalities has been extremely difficult to assess. Since the new millennium, cancer charities, the government and some dermatologists have argued that sunbeds caused the sharp rise of both melanoma incidence and mortality rates.^14^ Whereas other dermatologists insisted that it was caused by a modern lifestyle of greater sun exposure.^15^ Since the early twentieth-century, people in Britain have spent more time sun-tanning outdoors for leisure, and cheaper air travel has increased the popularity of overseas travel to hotter climates since the 1960s^16^; this decade matches the time when melanoma rates began to noticeably soar,^17^ whereas the sunbed ‘boom’ began in 1980.^18^ Clearly, this contentious health matter calls for historical study.

To situate the history of sunbed pathologising within other histories of moral panic and gendered addictions, the works of other tanning scholars need to be briefly unpacked. A brief contextualisation of the growing role of the mass media in public health will then support my novel focus on fictional media and ‘low-brow’ print press sources, such as the ‘Femail’ section of the *Daily Mail*, thus developing the history of public health approaches.

## Contextualising Tanning Culture, Situating ‘Sunbed Addicts’

Scholars of tanning and sunlight technologies have already historicised how tanned skin became both medicalised and commodified during the twentieth century; however, they did explore how this eventually led to the pathologisation of sunbed tanning from the late 1980s onwards. As these sunlight technology histories demonstrate, UV rays were—and still are—harnessed by healthcare professionals to cure many infections, skin diseases (i.e. tuberculosis, rickets and psoriasis), and mental health issues (i.e. Seasonal Affective Disorder and depression). But these rays can also be dangerous for skin and eyes. Despite growing medical concerns, most Western white people increasingly desired a tanned complexion throughout the twentieth century because it reflected more leisure time and travel; bronzed white skin eventually had become a marker of health and wealth.^19^ The growing medical and commodified use of artificial tanning devices, alongside this rise in tanning culture, led to the birth of the ‘sunbed’ industry in the late 1970s. Nonetheless, tanning historians have not yet fully addressed sunbeds in England. Nor have medical historians historicised how sunlight therapy morphed into the media–medical creation of ‘sunbed addiction’ that we know today.

Most medical historians agree that moral panics have emerged when the ‘wrong’ people began to consume. The state responds by stereotyping ‘wrong’ consumers as ‘irrational’ and framing the activity—such as ‘excessive’ nicotine, alcohol and food consumption—as irresponsible. Typically, these consumers were the already stigmatised working-classes, young women and especially mothers, who were ‘risking’ their conceiving potential or their children’s health. Consequently, the medical experts who discouraged these consumers (and the general public) acted on socio-cultural bias and moral sanctions.^20^ The fictional creation of the ‘immoral’ working-class sunbed user and later ‘tanorexic’ women supports these works by medical historians. Tellingly, sunbed users were often framed as the consumers of illegal drugs, alcohol and tobacco to ignite further moral judgement, perhaps aiming to discourage general sunbed use and encourage healthier lifestyles.^21^ Healthcare professionals also created a distinction between the medical ‘use’ and non-medical ‘abuse’ of UV technologies. This history of ‘tanorexia’ will provide a more novel ‘moral panic’ narrative by demonstrating how the media took the lead to transmit certain ‘health’ messages.

Medical historians have also shown that medical–media constructions of aesthetic-based ‘addicts’ were normally gendered. The extreme portrayals of ‘excess’ and ‘addiction’ were typically feminised, as ‘sufferers’ were deemed ‘weak’, ‘feeble-minded’ and lacking both self-discipline and control.^22^ By the late 1980s, sunbed usage was accessible to all and not restricted by gender, age, occupation, (dis-)ability or socio-economic background. Yet the ‘tanorexic’ stereotype was similar to those depicted in the histories of hysteria and eating disorders (particularly anorexia)—both were presented as a girl’s or women’s conditions,^23^ unless men, of course, were being framed as ‘homosexual’ or ‘metrosexual’.^24^

Most historians agree, such as Kenneth Lipartito and Adrian Bingham, that the mass media offers ‘an excellent primary source for exploring the history of media and communication’ as it provides a reflective narrative on political and societal events.^25^ Moreover, in Britain, the media played a greater role in delivering public health messages during the late twentieth century, demonstrating its growing authority in communicating health to ‘the public’.^26^ More recently, scholars have started publishing on how even fictional content influenced public understandings of health and everyday behaviours.^27^ An exploration of ‘tanorexia’ builds on these works by demonstrating how the media (in conjunction with the public), rather than medical experts and government, became the more important institution leading the debate on the dangers of sunbeds and the stereotypes of sunbed users. A more fine-grained analysis on both sunbed-related entertainment media and ‘low-brow’ print press coverage will demonstrate how the media influenced the direction of scientific research and medical conclusions.

As sunbeds became a widespread health concern of the working-class masses, the *Daily Mail* is a significant primary source for this article. The populist approach of this ‘tabloid’ incorporated a conservative political agenda and the target readers included the working-middle classes as well as the working-class. As a mass market newspaper, the *Daily Mail* also regarded itself as a quality middle market paper and therefore viewed itself closer to a broadsheet, contributing to its influence as an authoritative source of information for the public. Other than the *Sun*, the *Daily Mail* was the largest circulated newspaper and is therefore a valuable source that highlights popular social, cultural and political tensions caused by historical events and debates.^28^

More importantly, the *Daily Mail* targeted both men and women from the outset. It had one of the longest and largest female readership levels compared to all other newspapers and was even one of the first newspapers to provide features specifically for women. Since the early twentieth century, these features reflected ‘women’s consumer aspirations for … goods and lifestyles’.^29^ In 1968, the *Daily Mail* launched their ‘Femail’ section to attract even more women. It was edited by Shirley Conran, the author of the renowned 1970s *Superwoman* books on ‘household management’ for working women (and apparently men).^30^ Albeit leaning more towards conservative women, the tone of the ‘Femail’ section increasingly reflected liberal and therefore contradictory encouragements for women to be more ambitious, confident and independent. Yet for female readers, a sense of success was to be achieved by learning how to ‘better’ manage their bodies. The Femail section advised women on how to develop reportedly healthy, beautiful and fit bodies. This was often sold as a way for women to capture male’s attention within domestic and increasingly public spaces.^31^ As such, since the early 1970s, the ‘Femail’ section tried to reflect ‘women’s agency’ and ‘earning power’, while reflecting and feeding the growing middle-class preoccupation with health, diets, fitness, fashion and furnishing.^32^ During the 1980s and 1990s, the Femail page also published a noteworthy amount of medical, fitness and (‘bodytalk’) ‘scoops’. These ‘scoops’ have been overlooked by historians despite being an instrumental source of health and beauty information for a large number of everyday women in Britain; in 1992 alone, roughly 1.7 million *Daily Mail* papers circulated in Britain.^33^

As tanning has long been tied with ‘beauty’, ‘health’ and ‘fitness’, the *Daily Mail—*and the ‘Femail’ page in particular—were extremely vocal about ‘tanorexia’. In their anti-sunbed campaign pieces, the journalists acted as an opinion leader and influence, in a sense inventing ‘tanorexia’. Yet, most ironically, the *Daily Mail*, compared to all other newspapers at this time, most ardently supported alternative tanning industries (i.e. tanning lotions and pills) and, in fact, continued to recommend sunbed sessions to their readers.^34^ Late twentieth century women were expected to take pride in their appearance and follow the advertised fashion of a bronzed complexion, as this depicted health, fitness and beauty.^35^ Yet modern-day virtues of moderation discouraged ‘excess’, and women in particular were expected to consider how their behaviours may affect their long-term health.^36^ These women, like their nineteenth century predecessors, were expected to be compliant with expert advice by avoiding activities that risked their health, especially those that could reflect vanity.^37^ Evidently, the sentiment towards sunbed consumers in the *Daily Mail* was emblematic of the broader contradictory expectations of women’s bodies and social roles at the cusp of the 1990s. This exploration of a tabloid newspaper will be supplemented by examination of other popular media, such as a television programme.

## The Fictional Creation of the ‘Immoral’ Sunbed Consumer in the Media

During the late 1980s, as cancer fears grew, both television presenters and medical experts attempted to discourage the public from using sunbeds.^38^ Yet sunbeds remained within everyday private and public spaces, endorsed by beauty, health, fitness and fashion marketing. In 1989, the *Health Education Authority* launched a campaign to increase young women’s awareness of skin cancer. A survey-based study, conducted before and after the campaign, revealed that skin cancer awareness was already extremely high. The public’s awareness that sunbathing and sunbeds contributed to skin cancer did not discourage their tanning habits; ‘bronze [remained] beautiful’.^39^ While this study and campaign occurred, an ongoing avalanche of media broadcasts began to reflect and later intensify a sunbed-consumer moral panic. In the background of the medical profession’s and government’s bubbling anxieties about people’s persistent use of sunbeds, the media began to create and then reinforce a fictional sunbed stereotype.

At first, these sunbed consumers were depicted as working-class, young, vain and ‘immoral’ members of society. Soon after, consumers were depicted as ‘bimbos’, ‘barbies’, ‘gold diggers’ and later ‘evil stepmothers’ through the print press, a radio show, a television soap, a cartoon and later films. Across these media, the everyday sunbed consumer was satirically stereotyped as morally distasteful and disruptive: normally blonde, lazy, impulsive, selfish, cruel and self-destructive. By exploiting this stereotype, the act of using sunbeds was further stigmatised as frivolous, irrational and ignorant. In media representations, only the emotionally disconnected members of society indulged in sunbed use.

This rhetorical and visual culture—created and disseminated by both men *and women—*was misogynistic and derogatory. Fuelling moral panic was perhaps a response to the defiant sunbed consumers who were disinterested in the risks of skin cancer and refused to change their tanning habits. This may have been intended as an attempt to decrease skin cancer rates, aiming to improve the long-term health of the British public. Yet, this anti-sunbed coverage was delivered with the stigmatisation, condemnation and stereotyping of marginalised groups.^40^

Neither the government nor the medical profession was responsible for the creation and reinforcement of the original sunbed stereotype. Medical and government reports had simply stated that the main sunbed consumers were ‘young females’. But media reporters and creators knew entertainment and shock tactics would drive readership and viewers interest.^41^ The moral panic caused by the creation of this repellent stereotype may have discouraged people from admitting their sunbed use or, more importantly, seeking medical help if melanoma-suspect skin issues later emerged.^42^

The creation and development of the ‘immoral’ sunbed consumer within the popular media also reflected the political tensions of everyday public life in Britain. As a result of Thatcherite policies, the political, economic and subsequent consumer climate had drastically changed from the early 1980s to late 1980s and ‘yuppie’ culture (‘young upwardly mobile professional’) had reached household recognition by the end of this period. The ‘yuppie’ hallmark was an unapologetic attitude to personal success through the flaunting of ‘excessive’ and ‘irrational’ mass consumerism—presented as boastful and against the former British tradition of performing modesty when making money. These ‘yuppies’, both men and women, were said to originate from working-class backgrounds. They became a hated stereotype by those who remained poor and those who came from ‘old wealth’, founded from their middle-to-upper class backgrounds. When people saw ‘yuppies’ parading their money, it was perceived as a consequence of Thatcherite policies and subsequent individualism and social disorder.^43^

As sunbeds were no longer perceived as a middle-to-upper class consumption, they were soon framed as a symbol of grotesque vanity linked to ‘yuppies’, and the creation of the sunbed stereotype was consequently tied into the political backlash against those experiencing newfound self-made wealth and status. For instance, in a *Guardian* newspaper article titled ‘Loadsamoney making fun’ (May 1988), the sunbed, as an object, became an indication of an ‘immoral’ lifestyle when the term ‘immoral’ could not be explicitly said. In this article, the* Guardian* reporter satirised a fictional couple’s appearances, their household possessions, and their weekly routines to frame a frivolous and ‘party hard lifestyle’. The protagonist, Jason, was described as ‘a bit of a lad’, with a ‘gold earring’ and ‘golden highlights’. The couple had their own sunbed installed in the spare bedroom of their flat in North London and were offended when ‘middle-class snobs’ assumed they lived in a council flat. During their non-stop weekend parties, they did ‘poppers (amyl nitrate), a few bombers (amphetamines)’, coke and occasionally ‘smoked a little [heroin]’.^44^ Evident by the article’s title, the reporter was mocking the character and later novelty song ‘Loadsamoney’. Harry Enfield, a comedian and actor, had created and regularly performed the character ‘Loadsamoney’ in his television sketches for Channel 4’s *Saturday Live*. The character (and song) parodied a vulgar, unintelligent and flashy plasterer, with a cockney-accent, who waved wads of cash at those less fortunate. It personified 1980s working-class (but not middle-to-upper class) greed. This term quickly turned into a catchphrase and was regularly used to mock Thatcherism and the Thatcherite drive for the aspirational and successful working-class.^45^ In parliament, Margaret Thatcher even used the catchphrase to defend herself and the British economy. After 2 weeks in the ‘top 10’, the song remained in the British charts for 7 weeks during the late spring and early summer.^46^ When compared to most other tabloids, the more expensive and highly regarded *Guardian* was mostly read by educated, middle-class men.^47^ Through this article and others like it, middle-to-upper class readers were associating sunbeds with the drug-taking (and ‘excess’) by the working-classes who now had ‘loadsamoney’ to spend on ‘aspirational’ items and lifestyles.

In November 1988, a scriptwriter for radio drama—Anthony Minghella—used the ‘artefact’ of a sunbed, again, to depict the heartlessness of the heroine’s best friend in his radio play *Cigarettes and Chocolate*, as the sunbed tan could not speak for itself. In a discussion between radio scriptwriters for Radio 4’s (and the World Service’s) Globe Theatre season, Minghella explained that he chose his words carefully to ‘build artefacts’ and establish characters to radio listeners. He then explained that the protagonist, Gemma, was the ‘dumbest heroine since Kattrin in *Mother Courage*’.^48^ Gemma’s best friend was Lorna, a fellow ‘yuppie’. In one of Lorna’s monologues, she admitted to owning a sunbed, which she hid and secretly consumed in the middle of the night. This framed sunbed use as a shameful and guilt-ridden activity. Moreover, Lorna’s mother had committed suicide, and Lorna had used her inheritance money to create an ‘indulgence account’. She remorselessly confessed that she appreciated this money as it paid for her ‘hair streaked … a manicure … silk underwear … and the sunbed of course’.^49^ The following week, the *Financial Times* recounted this radio discussion about *Cigarettes and Chocolate*. Tellingly, the *Financial Times* was mostly read by wealthy middle-aged men and ‘traditional’ housewives during the 1980s.^50^ To middle-to-upper class radio listeners and newspaper readers (who used to be ‘responsible’ and ‘rational’ sunbed consumers), sunbed users were being presented as venal and uncaring.

Similarly, in a 20-part series called *Hollywood Sport* (1989), featuring on Yorkshire television,^51^ ‘everyday’ sunbed use was used to frame the only ‘immoral’ protagonist. The weekly series was based on the relationships between two married couples. Francesca (Jane Cunliffe) was ‘blonde, beautiful [and] bored’—she visited the sunbed, swimming and sports centre everyday. She was financially supported by her husband’s self-made business and was stereotyped as both self-absorbed and adulterous. This undesirable representation was accentuated by the stark contrast of the other wife, Claire (Andrea Gordon), who ‘ooze[d] good looks and charm’. Claire was married to Neil, her business and squash partner. Yet Claire’s dedication as a brilliant schoolteacher often ‘bruise[d] her husband’s ego’. *Hollywood Sports* was one of Britain’s first interactive viewer-controlled soap operas, and the first episode ended on a cliff-hanger with three options for audiences to call in and vote. The options included: a ‘passionate fling’ between Francesca and Neil; Neil rejecting Francesca because of his loyalty to Claire, or a secret meeting between Francesca and Neil. All these options presented Francesca as immoral, whereas Neil had one option to redeem his integrity.^52^ Moreover, Neil’s ‘bruised ego’ was presented as an acceptable excuse for an affair, directing the audience to empathise with him. Yet Francesca ‘cheating’ on her husband was unacceptable as she was financially supported by her husband. In all three viewer-controlled options, the sunbed consumer protagonist was disempowered and demonised as the most immoral character.

The following year, in January 1990, an article titled ‘…The full Bimbo teach-in’ appeared in the *Daily Mail’*s ‘Femail’ section. This ‘bimbo’ discourse demonstrated the media’s perpetual framing of the unethical sunbed stereotype into the early 1990s. The reporter described these ‘young women’ as ‘attractive but unintelligent or frivolous’. Their relationship statuses were depicted as always ‘available; the plaything of many a bored businessman, aspiring pop star—or even politician’. This article was apparently inspired by a court hearing, where a judge was confused about the differences between a ‘Bimbette, Bimbo and Ageing Bimbette’. This question, shocking both the legal prosecutor and defendant, supposedly led to a definition of these three ‘types’ of women. The *Daily Mail* used different categories, such as ‘Looks’, ‘Lifestyle’, ‘Hobbies’, and ‘Boyfriends’ to explain their differences. During their 20s, they all had ‘sunbed suntan(s)’. The reporter depicted the ‘bimbos’ as self-obsessed and against ‘commitment’. Yet they indulged in ‘expensive dinners … expensive clubs … gifts, credit cards and holidays offered’ by men. In later life, these ‘bimbos’ became alone. Their life aspirations were based on their appearances (such as ‘modelling’), which failed because they aged. The *Daily Mail* framed these women as irrational money-leeching beauty consumers, who would reap poor, lonely, boring and unfulfilling moral consequences for their ‘self-absorbed’ life decisions.^53^

**Fig. 1.**
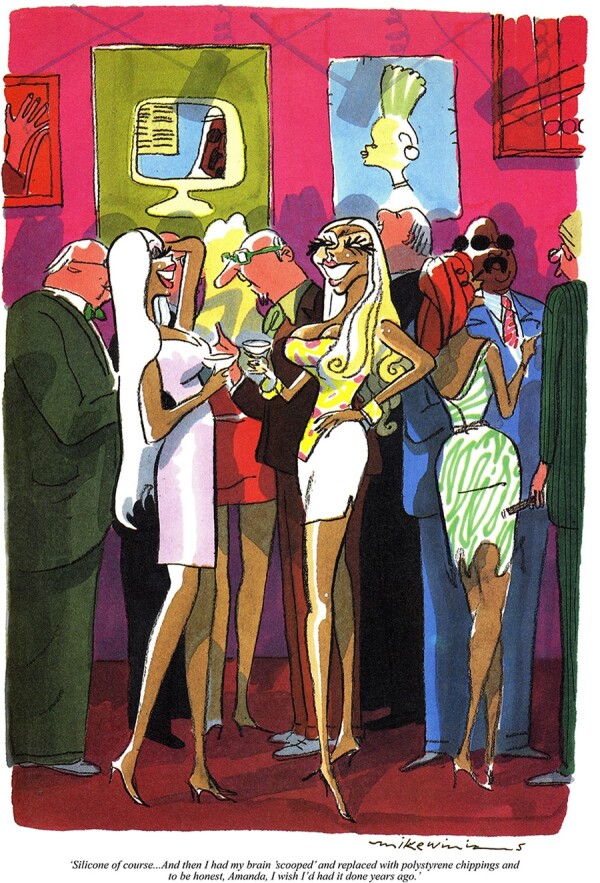
‘Bimbo’ stereotype cartoon in *Punch* magazine, May 1990 *Source:* Reproduced with permission from Punch Cartoon Library/TopFoto.

In May 1990, a few months after the ‘Bimbo’ *Daily Mail* article, a *Punch* cartoon drawn by Mike Williams depicted a more visually shocking ‘suntanned bimbo’ stereotype as shown in Figure 1.^54^*Punch* was a long-established British weekly magazine of humour, often using cartoons to satirise political and social affairs.^55^ The setting of this particular cartoon was a glamorous soiree in an art gallery. The cartoon’s centrepiece were two young women in front of a crowd of people, both small talk and cocktails flowing. The men were old, bald and dressed in suits and spectacles—one with a cigar in his hand. These wealth-reflecting men were accompanied by their ‘gold digging’ girlfriends and ‘escorts’. The two ‘Barbie doll’ lookalikes wore stilettos and spider-like eyelashes. Their tans accentuated their bright white teeth as they grinned at each other and their long bleached blonde hair reflected high maintenance. The women’s chiselled faces and pneumatic figures suggested plastic surgery and breast implants. The cartoonist presented the women as brainless ‘arm candy’, highlighted by the cartoon’s caption:

Silicone of course … And then I had my brain ‘scooped’ and replaced with polystyrene chippings and to be honest, Amanda, I wish I’d had it done years ago.

This dialogue emphasised that these women were artificial, shallow and vacuous. Both women were drawn in a way that accentuated and directed the viewers’ focus to their breasts. The bodily representation of these women radiated vanity and confidence. This cartoon insinuated that sunbed ‘bimbos’ were not intelligent enough to self-fund their ‘indulgent’ beauty regimes and lifestyles. Instead, these women lived precariously and unproductively through the financial support of older and richer men.^56^ This stereotyping continued after the 1990s, evident by the villain from *Cinderella Story* (2004)—Fiona, the evil stepmother and ‘vain gold digger’. Fiona stole her stepdaughter’s inheritance and college fund to purchase both cosmetic surgeries and her baby pink sunbed, which she regularly used in her sunny Californian backyard—this framing itself creating an association of tastelessness through a conspicuous and pointless consumption.^57^

In medical reports at the time, the women who regularly used sunbeds were recognised as affluent,^58^ yet in all fictional accounts this wealth was unearned. The apparent ‘bimbos’ acquired wealth from other financial sources, mainly wealthy businessmen or the deceased.^59^ Sunbed use was being presented as a typical activity of ‘unintelligent’ women. In the media, sunbed use was not mentioned in any lifestyle accounts of ‘wealthy’, ‘successful’, ‘intelligent’ or ‘diligent’ women from the late 1980s onwards. This was not because affluent women had refrained from using sunbeds but rather sunbeds no longer reflected moral worth. Instead, by the 1990s, sunbed consumption was used to frame women who were judged to have repellent personalities and lifestyles—even in fictional novels.^60^ These ‘repellent’ women were entering male-oriented careers and public spaces, and were apparently ‘selfishly’ exercising their growing spending powers, and embodying confidence and independence, which was now associated with a sunbed tan.

Although the government and medical officials had held back from creating and stigmatising stereotypes so far, the media’s translation of these anti-sunbed ‘health messages’ had clearly arrived with gender, class, age and sexuality-bound judgements. Moreover, this coverage was indirectly endorsed by the confirmed link between skin cancer and sunbeds, now widespread in the media. The media had presented the immoral sunbed stereotype as an irresponsible, lazy, narcissistic and self-destructive consumer, who took advantage of others through their lifestyle choices. Nonetheless, these scare tactics failed. In part, they failed because anti-sunbed messages were competing with a decade-long reinforcement, alongside constant visual messages, that sunbeds were desirable, ‘healthy’ and ‘safe’. Consequently, consumer attitudes and everyday rituals proved difficult to change. After the early 1990s, consumers continued to use sunbeds but went to greater efforts to conceal their consumption because of these conflicting messages.^61^

Although the 1980s concluded without any hint that sunbeds were addictive, or that sunbed use was disordered, the growing secrecy behind sunbed use would make the pathologising of consumers easier. Secret sunbed use would fit the early 1990s’ ‘addiction’-criteria, which medical experts were developing to explain other self-destructive consumptions. Like the histories of alcohol, tobacco and drug addiction, blaming individual sunbed consumers was one-sided as it overlooked the environmental, commercial and socio-cultural pressures driving people to consume. For instance, the expansion in manufacturing, communication and transportation technologies—which assisted persistent mass advertising, cheaper pricing and accessibility—influenced an increase in overall sunbed consumption.^62^ Moreover, these external factors were not isolated driving points. Instead, they created an interacting web of influence that promoted tanned skin and contributed to ‘excessive’, or more so regular sunbed use.

## The Media–Medical Creation and Circulation of ‘Tanorexic’ Women

After the early 1990s, newspapers confidently asserted that sunbeds were a key factor for the growth in skin cancer rates. Yet publicity campaigns against UV-A sunbeds were still failing to discourage sunbed consumers. Medical experts, such as dermatologist Dr David Shuttleworth, argued that sunbed use continued because they remained in health-associated environments (i.e. gyms), and their providers refused to remove them and their advertisements that promised enhanced health.^63^ Unlike the products of other cancer-causing industries, the government and medical authorities could not effectively intervene as sunbeds could not yet be regulated. Nor could outdoor natural tanning be stopped. As sunbeds could not be removed from health spaces (except through fictional portrayals), reporters focused even more on discouraging individual sunbed users. Newspapers—mainly the *Daily Mail—*continued to shift the sunbed tan away from ‘fit’ and ‘healthy’ bodies onto ‘sunbed addicts’, now endorsed by medical authorities. If the media framed ‘tanorexia’ as affecting one particular demographic group, the disorder would be easier to disseminate and discourage through the national press.^64^

In general, a wider trend was emerging as both medical experts and the media were increasingly using medical theories, such as the ‘addiction’ rhetoric,^65^ to both describe and explain women’s increasingly ‘irrational’ and ‘dangerously obsess[ive]’ behaviour towards many beauty consumptions—particularly sunbeds, cosmetics and cosmetic surgery.^66^ Moreover, reporters were offering more educational platform for ‘experts by experience’ to share their own opinions, emotions and experiences of everyday beauty, health and lifestyle affairs with other people. They wanted to attract their readers’ attention—particularly through the widely read Femail section of the *Daily Mail*. This reflected and contributed to the rise of the consumers’ voice and confessional culture during the early 1990s.^67^ In turn, people felt more responsibility to intervene, and try and change other people’s lifestyles, as they now had the resources to do so. This blurred the boundaries between ‘expert’ and ‘public’, and private and public spheres further.

A detailed evaluation of the new ‘sunbed addiction’ model also untangles several rival narratives of 1980s and 1990s England. As I previously mentioned, confident and young working-class women were exercising their growing independence and financial freedom, and thus modifying their bodies to reflect what they considered was healthy. For those against these women’s new confidence, sunbed consumption represented a crisis in morality. But for others, the sunbed addiction narrative actually helped people in develop an understanding of everyday tanning behaviours—even for those doing the tanning themselves. The growing fears of melanoma, now linked to sunbeds, was another reason why ‘sunbed addiction’ was taken seriously. The ‘addiction’ model, however, overlooked the increasing commercial and visual pressures in the media, which pressurised both women and men to develop certain body appearances. In particular, the 1990s boom of the fake tan industry, combined with constant discussions about tanned skin in the print press, was bound to trigger an anxious preoccupation with both pale and tanned skin. Sunbed ‘addiction’ was similar to other conflicting narratives at the time, like those on drug-taking, which were circulated to explain why young people were ‘excessively’ consuming in ‘reckless’ behaviours.^68^ Moreover, the association of ‘tanorexia’ with ‘anorexia’, and ‘tanning addiction’ with medically verified biological and psychological addictions (i.e. nicotine, alcohol and illegal drugs), would have further pathologised sunbeds and their consumers.^69^

From 1991 onwards, both male and noticeably female reporters depicted female ‘sunbed addicts’, under the catchier and more provocative term ‘tanorexics’, across national newspapers. An evaluation of three *Daily Mail* ‘femail’ exclusive(s)’ and a ‘special report’, published in May 1991 and July 1992, demonstrates how several ‘tanorexic’ case studies intensified the immoral sunbed consumer stereotype, as described in the previous section, into a pathological ‘sunbed addict’. An overview of these case studies will expose who the reporters and medics framed as sunbed addicts; what these consumers were said to prioritise in their lifestyles; and why, how often and in what way these ‘tanorexics’ were said to use sunbeds. Finally, I will demonstrate how the reporters created a moral message to try and scare women into following anti-sunbed health warnings. These reporters pathologised ‘tanorexic’ women as vain, deranged, out of control, excessive and self-destructive.

In one *Daily Mail* article (May 1991), the reporter portrayed the new ‘addicts’ as ‘smart, confident’, ‘high-achieving and successful’ women; women who could (now) afford their ‘fix’. Again, these women were framed as having disconcerting personalities and undesirable lifestyles. The reporter then described these women’s beauty addiction cycle. First, the addict was desperate, furtive and excited. Next, she felt ‘tremendous relief followed by guilt when the substance ha[d] finally been purchased’. Apparently, these beauty addicts were no longer older women in their thirties, whose vanity was funded by their husbands. Instead, these young women were in their twenties and now earned their own incomes. The reporter remarked that these women spent their money ‘irrationally’ on beauty consumptions at the expense of more important priorities. These priorities included ‘their homes, their husbands, their families, their jobs and their social lives’, which they could not commit to until they were aesthetically ‘perfect’.^70^ Women were being condemned for exercising their growing financial independence and spending powers beyond their traditional household and motherly expectations.^71^

A year later (July 1992), another *Daily Mail* article asserted again that ‘tanorexics’ were typically white women in their twenties; however, only three out of six female interviewees were in their twenties (Table 1).^72^ In this article, the seven ‘tanorexic’ profiles consisted of six women and one man. An example of a ‘tanorexic’ man was rare. In 1994, the overall ratio of men to women who had used a sunbed was seven to eleven.^73^ Yet female sunbed users were significantly overrepresented in both the media and scholarly texts as health experts were more likely to identify a psychological problem of excess in female patients.^74^ Although the *Daily Mail’*s unique inclusion of a ‘tanorexic’ man somewhat balanced the discussion, the man’s biography consisted of two superficial paragraphs, which contrasted to the women’s in-depth profiling. As these two small paragraphs featured at the bottom of the two-page article they could be easily overlooked by readers. This man also had a feminised job as a ‘florist’. The readers, like the journalists, would no doubt focus on the ‘tanorexic’ women. As cancer was perceived as a ‘white woman’s nemesis’ in Western culture throughout the twentieth century, brown and black people were excluded from all melanoma-related media coverage and medical studies.^75^

**Table 1.** ‘Tanorexic’ profiles in a *Daily Mail* article, July 1992

	Surname	Gender	Location	Age	Occupation/Status	Skin type
**1**	Wieck	Female	Windsor	19	Beauty therapist	‘Olive skin’ (Sri Lankan mother)
**2**	White	Female	London	23	Secretary	White
**3**	Sayles	Female	Berkshire	25	Hairdresser	White
**4**	Catchpole	Female	Dorset	27	Telesales controller	‘Red hair’ and ‘pale, Anglo-Saxon’ skin
**5**	Brimmell	Female		37	Divorcee	‘Fair-skinned redhead’
**6**	Button	Female		44	Housewife	White
**7**	Nunn	Male	Peterborough	44	Florist	‘Fair skin’

Unsurprisingly then, six out of seven of these ‘tanorexics’ were explicitly categorised as white, and were said to be most at risk from developing melanoma. The reporter emphasised that three, in fact, had ‘fair skin’; two of whom were ‘redheads’. This stressed to readers that their sunbed habits were even more hazardous and senseless. The article also stated that ‘tanorexics’ were usually ‘models’ or in careers where their appearance was ‘important’. Both employers and clients would expect these women to maintain an aesthetic of ‘respectability’ when performing their social roles and jobs (as a housewife, divorcee, beauty therapist, secretary and hairdresser). Moreover, these working women were not in a privileged position to challenge these expectations. Therefore, with the controversial yet continued fashion for tanned skin, the pressures felt by these women to maintain a tanned complexion was not ‘irrational’.^76^

Nonetheless, these newspaper reporters, supported by healthcare professionals, framed ‘tanorexics’ as impulsive consumers who were out of control. In two Femail exclusive ‘special report(s)’ on ‘beauty addicts’ and ‘beauty slaves’ from May 1991, the reporters told the public that these ‘cosmetic junkies’ had ‘neurosis’, ‘compulsions’ and ‘obsessions’, which explained their sunbed use.^77^ These descriptions were repeated in the July 1992 *Daily Mail* article to describe ‘tanorexics’ in more depth. In this article, Dr Misra claimed that ‘tanorexia’ was a ‘psychological addiction’ tied to a sense of self-esteem. He remarked that a sunbed addict’s vanity overcame her fear of cancer. His patient, Catchpole, concurred that her behaviour was ‘madness’. As a ‘fanatic’, she was ‘utterly hooked’ and ‘obsessed’ with sunbeds.^78^ As a heavy smoker, Catchpole also believed she was ‘immune’ from skin cancer and that sunbed concerns were ‘superfluous’. Whereas Brimmell rationalised that she had bought a sunbed because it did not burn her unlike the sun. For Wieck, sunbeds made her skin ‘feel healthier’ and ‘nourished’. White used sunbeds because she could not afford a holiday and the ‘psychological lift’ cured her depression. Sayles also believed that sunbeds were ‘less harmful’ than the sun, and the heat alleviated her neck problem. During the 1980s, these motives were advertised as ‘rational’ reasons why people should use sunbeds.^79^ Yet when these women repeated these reasons to defend their sunbed use, both reporters and medics used this defiance to pathologise them even further. Like many people in Britain at the time, these women were unphased by the growing ‘endless stories’ on cancer.^80^ People were often in denial as they perceived cancer as an ‘invisible’ and mysterious illness that affected others and not themselves.^81^

Although the *Daily Mail* reporter started to acknowledge the ‘psychological benefits’ of sunbeds—as ‘countless surveys’ had confirmed that sunbed tans made consumers feel ‘slimmer, more sexually attractive and therefore more confident’—the reporter then countered this validation by emphasising that these women were just insecure, vain and had low self-control.^82^ Both reporters and medical experts argued that these women’s ‘excuses’ were unjustified.^83^ Yet, in social and working spaces, aesthetic imperfections were also stigmatised and presented as an economic disadvantage, especially for women.^84^ This combination created a moral and social contradiction in the expectations of women’s health and bodies—typical of the *Daily Mail’*s Femail section. Nonetheless, the pathologising of their guilt-ridden sunbed behaviour was a typical public health approach to discourage consumption.^85^

These sunbed users were also presented as ‘addicts’ because ‘regular’ sunbed use was open to interpretation—it could mean daily, weekly or seasonally. The duration or protective measures that these ‘addicts’ undertook did not matter.^86^ Some of these women even argued that they used sunbeds responsibly. Yet, these newspaper articles framed consumer acceptance of the ‘risk’ as irresponsible.^87^ Moreover, these reporters were disregarding 1980s sunbed advertisements in which ‘everyday’ sunbed use was both encouraged and presented as harmless, if not health-enhancing.^88^

To emphasise that their ‘fix’ was unreasonable, these women’s sunbed use was presented as both absurd and secretive. In a section captioned ‘Bizarre’ from the first *Daily Mail* article (May 1991), the reporter ridiculed a bride for wanting a beautician in London to open her salon on a Saturday morning before her wedding day.^89^ This advice directly contradicted another *Daily Mail* reporter two years later, who advised a course of sunbed sessions if a soon-to-be bride was unhappy with her ‘pale’ complexion.^90^ This secretive and ‘guilty’ use of sunbeds continued into the mid-1990s. A model remarked that people still used sunbeds but denied it in public because they did not want to be stigmatised and condemned.^91^ The reporters and medics pathologising this guilt were overlooking the mixed messages that continued to glamorise bronzed complexions in the print press.

## ‘Death’ and Shame: Moral Messages to Discourage ‘Sunbed Addicts’

During the early 1990s, the media presented ‘sunbed addiction’ as ‘costly’ and life-threatening—a burden on the National Health Service, the taxpayer and the public. The reporter and medical experts also remarked that sunbeds contributed to ‘28,000 cases of skin cancer a year and 1,500 deaths’. Like the rest of the *Daily Mail’*s coverage on sunbeds, dermatologist Dr John Hawk provided another mixed message. He suggested that a cosmetic sunbed tan was deliberate skin damage, whereas the public outside in the sun were ‘at least … enjoying life’. Sunbeds caused ‘itching, irregular freckling … prickly heat … dry skin … mild sunburn and premalignant moles’ and also ‘skin fragility syndrome—nasty crusts, scabs and blisters’. Yet, reportedly, these were not as ‘insidious’ as the tanorexia ‘syndrome’.^92^ This specific focus on the aesthetic damage of sunbeds reflects their attribution of vanity to these consumers, ignoring the users’ experienced benefits.

Nonetheless, the term ‘tanorexia’ still remained relatively uncommon during the early 1990s. The evidence to conceptualise sunbed use as a widespread addiction was sparse. This changed in 1994. The melanoma deaths of two women from Newcastle marked a turning point. All newspapers reported that these ‘sunbed’ deaths were the first cases in England to be directly linked by a doctor. This immediately strengthened the medical profession’s authority to comment on sunbed use. In these newspapers, dermatologists, such as Peter Farr and John Hawk (who regularly featured in sunbed related press), narrated the fatalities in a way that would significantly increase public fears. Farr and Hawk, who worked together for the British Photodermatology Group, stated that these two deaths were entirely caused by sunbeds.^93^ They claimed that one of these two ‘young’ women had only been on one foreign holiday and neither had ‘sunbathed topless or nude’; therefore, they were ‘fairly confident that natural sunlight played no role at all’. One of these ‘young’ women was, however, in her forties. Nonetheless, the reporter presented these dermatologists as ‘leading skin experts’ who forthrightly ‘condemn[ed] regular’ sunbed use. Dr Farr claimed that these deaths were the ‘tip of the iceberg’ of sunbed related casualties as they were certain that the skin cancer process took several years. The ‘worse offenders’ were those who used sunbeds ‘indiscriminately’ at home.^94^ Such unmonitored household use was now constructed as a feckless consumption of working-class people, not rational and affluent consumers.^95^ Calling these consumers ‘offenders’ also framed their consumptions as a criminal activity—a typical trend of the addictive trope.^96^

The timing of these sunbed-linked deaths created a strong public response. First, skin cancer in Western culture was often headline news in the 1990s.^97^ Second, the deaths of two white, ‘young’ women in the media would more powerfully evoke sympathy compared to other demographic groups.^98^ This heightened further the public fear and moral panic associated with sunbeds. In a response to these deaths, England’s biggest sunbed hire group, HSS Hire Shop, abandoned the launch of their new sunbeds across 170 stores.^99^ A month later, a television programme highlighted the dangers of sunbeds and ‘tanorexia’.^100^ The ‘tanorexic’ then began to regularly appear in newspapers (unsurprisingly mainly through the *Daily Mail* and *Guardian*), magazines and more significantly on national television during the mid-1990s. This coverage was typically accompanied by psychologists and dermatologists, who confirmed both the short-term torment and long-term fatality caused by the sunbed ‘condition’.^101^ Even a study published in the *British Medical Journal of Dermatology* in 1997, titled ‘Why do young women use sunbeds?’, demonstrated how psychologists were endorsing a gendered ‘tanorexic’ stereotype.^102^ At the end of the 1990s, the British Imperial Cancer Research Fund reinforced sunbed addiction as a women’s condition. They claimed that ‘1 in 4 women suffer[ed]’ from ‘tanorexia’, and that these ‘addicts’ used sunbeds ‘more than once a week’.^103^ Tanorexia had become legitimised as a psychological addiction that primarily affected women.

Collectively, media agents, medical experts and the general public had played a strong part reinforcing ‘sunbed addiction’ as a gender-specific, life-threatening condition. Depictions of ‘sunbed addiction’ had spread from national newspapers and magazines to mainstream television, endorsed by healthcare professionals, to reach a wider audience. Newspaper journalists also began to use the term ‘sunbed tan’ to derogatively describe and disempower men and women. Media reporters may not have been able to distinguish between a sunbed and natural tan, yet they used the term to create shame. To this day, the shameful ‘sunbed tan’ is still associated with young, white and working-class women from underprivileged urban regions in Britain.

## Conclusion

This microhistory of the sunbed consumer builds on the long-established narratives of working-class moral panic and gendered addictions; however, it has done so uniquely through an evaluation of typically trivialised—but far reaching and influential—entertainment media and the *Daily Mail*, demonstrating the value of popular culture for historical research. This novel approach highlighted several political, economic and socio-cultural tensions within contemporary Britain, such as the Thatcherite backlash towards working-class consumerism, ‘yuppie’ culture and women’s increasing financial and bodily independence and freedom. It also demonstrated how the media (through the invention, development and reinforcement of the sunbed stereotype turned ‘addict’) reigned supreme in discouraging sunbed use—even if these ‘health messages’ were both unintentional and subliminal. The constant mass media circuits of sunbed consumer stereotypes also illustrated the blurring of public health research and popular culture in media-based health messages. Even in 2019, during a parliamentary discussion at Westminster Hall, sunbeds were incessantly described as an irrational and ‘vain’ activity in an attempt to ban them from the UK.^104^ Clearly, these typically overlooked media sources, including the ‘Femail’ section of the *Daily Mail*, have powerfully influenced public understandings of health ‘condition(s)’, alongside medical experts, industry and policy interpretations. As such, medical historians should evaluate these sources to uncover more late twentieth-century histories of women’s everyday health.

In terms of working-class moral panic, this article demonstrates how the media can create a representation of consumptions, possessions and everyday rituals to frame people’s moral worth. Framing cosmetic sunbed use as a feminine, egotistical and shameful consumption, which lacked self-discipline, was perhaps intended as a preventative strategy against the rise of skin cancer. The shame was meant to pressurise those who continued to use sunbeds—and those who were considering sunbed use—into compliance with health advice. Yet medical experts did not acknowledge the cultural bias of class-based and gendered expectations. Women, particularly those at potential child-rearing ages, were framed in the media as narcissistically and senselessly ruining their lives by irresponsibly draining societal resources (i.e. the doctors’ time both checking and removing skin cancer) and thus ruining the lives of others in the community. Such condemnation of ‘irrational’ behaviour reflects a historically renowned bias regarding women’s consumption. Society expected ‘moral’ women to both provide and raise children, as this was often presented as a women’s main contribution to wider society. Therefore, women were more shamingly presented as selfish for ‘indulging’ in self-destructive behaviour than equally self-indulgent men.

These gendered sunbed stereotypes also extend the histories of women and ‘metrosexual’ men being criticised for ‘vain’ consumptions. Although a suggestion or visual depiction of ‘sunbed addicted’ men rarely featured within media or medical coverage, it was occasionally deployed to mock a man’s ‘metrosexual’ tendencies, both emasculating and feminising them. Yet women’s sunbed use was regularly framed derogatively and pathologically, even by themselves. However, as ‘sunbed addiction’ was reinforced as a woman’s condition, men were less likely to interpret their sunbed consumption as a problem. Moreover, medical experts were overlooking the cultural expectations and greater bodily pressures placed on women in the media to be aesthetically desirable; women’s culturally accepted openness about their own beauty routines and tanning habits during the 1990s, unlike men, likely contributed to young, white women being more open about their sunbed use, and therefore more easily framed as a ‘sunbed addict’. White women often explained that a tanned complexion, albeit controversially, continued to be fashionable in most public spaces. White women were expected to have a tan to look ‘healthy’ and ‘attractive’ in most social roles (from domestic spaces to the workplace). These sunbed stereotypes then influenced which participants were chosen in future sunbed studies as healthcare researchers typically chose young white women.^105^ The stigmatisation of ‘tanorexics’ during the early 1990s was also in trend with the rise of addiction theories in medical settings to explain ‘irrational’ consumptions.

The gendering of a health behaviour and medical identity, in this case the ‘adolescent female sunbed addict’, perhaps weakened sunbed preventive education. Medical advice was more likely to be ignored as a ‘tanorexic’ stereotype was inappositely emphasised. In 1999, both the HEA and *The Times* worryingly estimated that approximately 3 million people in Britain continued to use sunbeds every year.^106^ The highly biased immoral depiction of sunbed ‘addicts’ instead encouraged some people to develop more secretive behaviours towards sunbed use,^107^ and men became less likely to seek medical advice on resulting skin conditions, in time for more effective treatment. Melanoma mortality rates were in fact higher in affluent men than in women in England during the mid-1990s.^108^ Even into the new millennium, the constant medical reinforcement of ‘tanning addiction’ has done little to discontinue this trend.^109^

## Footnotes

1 B. L. Diffey, ‘Use of UV-A Sunbeds for Cosmetic Tanning’, *British Medical Journal of Dermatology*, 1986, 115, 67–76; A. J. Swerdlow *et al.*, ‘Fluorescent Lights, Ultraviolet Lamps, and Risk of Cutaneous Melanoma’, *British Medical Journal*, 1988, 297, 22.

2 For a history of sunbeds between the late 1970s and early 1980s, including middle-to-upper class sunbed consumption and general changes in skin cancer concerns, see Fabiola Creed, *The Rise and Fall of the Sunbed: Tanning Culture from Fad to Fear* (Montreal: McGill-Queen's University Press, forthcoming).

3 Patsy Westcott, ‘I’d Rather Be Wrinkled and Brown than White and Smooth’, *Daily Mail*, 21 July 1992, 24–25; Steve Tooze, ‘Tanorexia’, *Daily Mail*, 16 May 1996, 44–45; Mandy Francis, ‘A Day in the Life of a Sunbed’, *Daily Mail*, 16 March 1999, 40–41. Although ‘sunbed addiction’ did not appear in the American Diagnostic and Statistical Manual of Mental Disorders (DSM) IV, many psychologists later argued that it was another form of ‘substance dependence’, and they correlated it with the criteria from the DSM-IV, Carolyn Heckman and Sharon Manne, *Shedding Light on Indoor Tanning* (Switzerland: Springer, 2012), 109–16; American Psychiatric Association, *Diagnostic and Statistical Manual of Mental Disorders*, 4th edn (Washington: American Psychiatric Association, 1994), 17–19, 181.

4 ‘Nicola Roberts: The Truth About Tanning’, BBC 3, 4 February 2010; ‘Loving the Tan?’, BBC2, 21 August 2010; ‘Hayley: Call Centre’s Tanning Addict’, BBC One Wales, 24 March 2016; ‘Tantastic: 50 Shades of Orange’, Channel 5, 2 June 2016; ‘True North, Series 13, Tanorama’, BBC One, 17 May 2021.

5 Glenn Collins, ‘Metropolitan Diary’, *New York Times*, 2 July 1980, 2; Scott Harris, ‘TANOREXIA: Having a Golden Tan can become a Compulsion for Some Salon Patrons’, *Los Angeles Times*, 25 August 1989, 2.

6 As the term ‘sunbed addiction’ was preferred in medical journals, the term ‘tanorexia’ only appeared in professional literature in the mid-2000s, C. Murray and E. Turner, ‘Health, Risk and Sunbed Use: A Qualitative Study’, *Health Risk Society*, 2004, 6, 67–80.

7 Westcott, ‘I’d Rather Be Wrinkled and Brown than White and Smooth’, 24–25; Anon., ‘Girl of 8 Are Dying for Tans on a Sunbed’, *Daily Express*, 26 June 2004, 17.

8 In 2012, American scholars of Public Health and Skin Cancer Prevention, Joel Hillhouse and Rob Turrisi, both correspond that the emergence of medical journals on sunbeds dramatically increased from 1992 onwards, Joel Hillhouse and Rob Turrisi, ‘Chapter 4: Motivations for Indoor Tanning: Theoretical Models’, in Heckman and Manne, *Shedding Light on Indoor Tanning*, 70.

9 B. Fiala, M. Kopp and V. Gunther, ‘Why Do Young Women Use Sunbeds? A Comparative Psychological Study’, *British Medical Journal of Dermatology*, 1997, 137, 950–54; Heckman and Manne, *Shedding**Light on Indoor Tanning*, 58.

10 Naomi Marks, ‘TV: Keeping Up Appearances’, *British**Medical**Journal*, 2003, 326, 770; Jack E. Henningfield, Christine A. Rose and Mitch Zeller, ‘Tobacco Industry Litigation Position on Addiction: Continued Dependence on Past Views’, *Tobacco Control*, 2006, 15, iv33; ‘Skin Cancer Update’, *Dermatology Nursing*, 2007, 19.4, 390–92; K. Diehl *et al.*, ‘First Evaluation of the Behavioral Addiction Indoor Tanning Screener (BAITS) in a Nationwide Representative Sample’, *British Medical Journal of Dermatology*, 2018, 178, 176–82.

11 R. Homung and S. Poorsattar, ‘Tanning Addiction: The New Form of Substance Abuse’, Skin Cancer Foundation, 2013.

12 Mackenzie R. Wehner *et al.*, ‘Indoor Tanning and Non-Melanoma Skin Cancer: Systematic Review and Meta-Analysis’, *British**Medical**Journal*, 2012, 345, e5909.

13 John Buchan and Dafydd Roberts, *Pocket Guide to Malignant Melanoma* (Hoboken: Blackwell Science, 2000), vi.

14 Cancer Research UK, *Policy Statement: Sunbeds*, 2009; House of Commons Library, (January 2010), *Sunbeds (Regulation) Bill*, Bill 19 of 2009–10, Research Paper 10/07, 27, 6; M. Boniol *et al.*, ‘Cutaneous Melanoma Attributable to Sunbed Use: Systematic Review and Meta-Analysis’, *British**Medical**Journal*, 2012, 345, 4.

15 Buchan and Roberts, *Pocket Guide to Malignant Melanoma*, 2.

16 Tania Woloshyn, *Soaking Up the Rays: Light Therapy and Visual Culture in Britain, c. 1890-1940* (Manchester: Manchester University Press, 2017); Bill Cormack, *A History of Holidays, 1812-1990* (London: Routledge and Thomas Cook Archives, 1998), 108, 112.

17 Reinhard Dummer *et al.*, *Skin Cancer: A World-Wide Perspective* (Switzerland: Springer, 2011), 14; Buchan and Roberts, *Pocket Guide to Malignant Melanoma*, 1.

18 Another explanation for the rise in recorded incidences was the advancing age of the population, the major improvements in skin cancer detection technology and more frequent examinations by dermatologists, Thomas Rustemeyer *et al.*, eds, *Kanerva’s Occupational Dermatology*, 3rd edn (Switzerland: Springer, 2020).

19 Woloshyn, *Soaking Up the Rays*; Simon Carter, *Rise and Shine: Sunlight, Technology and Health* (Oxford: Berg, 2007); Daniel Freund, *American Sunshine: Diseases of Darkness and the Quest for Natural Light* (Chicago: University of Chicago press, 2012); Devon Hansen, ‘Shades of Change: Suntanning and the Twentieth-Century American Dream’ (unpublished PhD thesis, Boston University, 2007); Sally Romano, ‘The Dark Side of the Sun: Skin Cancer, Sunscreen, and Risk in Twentieth-Century in America’ (unpublished PhD thesis, Yale University, 2006); Kerry Segrave, *Suntanning in 20th Century America* (Jefferson: McFarland & Company, 2005); Two psychologists have published the only book dedicated to indoor tanning machines, Heckman and Manne, *Shedding Light on Indoor Tanning.*

20 Virginia Berridge, ‘Constructing Women and Smoking as a Public Health Problem in Britain 1950–1990s’, *Gender & History*, 2001, 13, 328–48; Virginia Berridge, Rachel Herring and Betsy Thom, ‘Binge Drinking: A Confused Concept and Its Contemporary History’, *Social History of Medicine*, 2009, 22, 597–607; Alex Mold, ‘From the Alcoholic to the Sensible Drinker: Alcohol Health Education Campaigns in England’, in *Balancing the**Self* (Manchester: Manchester University Press, 2020), 88; Alex Mold, 'Consuming Habits: Histories of Drugs in Modern Societies’, *Cultural and Social History*, 2007, 4, 263–65; Sander L. Gilman, *Obesity: The Biography* (Oxford: Oxford University Press, 2010); Peter Stearns, *Fat**History: Bodies and**Beauty in the**Modern**West* (New York: NYU Press, 2002); Roberta Bivins, ‘Immigration, Ethnicity and “Public” Health Policy in Postcolonial Britain’, in Catherine Cox and Hilary Marland, eds, *Migration, Health and Ethnicity in the Modern World* (London: Palgrave Macmillan, 2013), 126–50; Allan M. Brandt and Paul Rozin, eds, *Morality and**Health* (Hove: Psychology Press, 1997).

21 Helen Chappell, ‘Loadsamoney Making Fun: Third Person’, *Guardian*, 11 May 1988, 16; Fiala, Kopp and Gunther, ‘Why Do Young Women Use Sunbeds?’, 950.

22 Virginia Berridge, *Demons: Our Changing Attitudes to Alcohol, Tobacco, & Drugs* (Oxford: Oxford University Press, 2013); David T. Courtwright, *Forces of Habit. Drugs and the Making of the Modern World* (Cambridge: Harvard University Press, 2001); Julia Skelly, *Addiction and British Visual Culture, 1751-1919: Wasted Looks* (Surrey: Ashgate Publishing Ltd, 2014); Julia Skelly, *The Uses of Excess in Visual and Material Culture, 1600-2010* (Milton Park: Routledge, 2018); David Serlin, Liping Bu and Lisa Cartwright, *Imagining Illness: Public Health and Visual Culture* (Minneapolis: University of Minnesota Press, 2010); Jessica M. Parr, ‘Obesity and the Emergence of Mutual Aid Groups for Weight Loss in the Post-War United States’, *Social History of Medicine*, 2014, 27, 768–88.

23 Andrew Scull, *Hysteria*: *The Biography* (Oxford: Oxford University Press, 2009); Joan Jacobs Brumberg, *Fasting Girls: The History of Anorexia Nervosa* (Cambridge: Harvard University Press, 2000); Joan Jacobs Brumberg, *The Body Project: An Intimate History of American**Girls* (New York: Vintage, 1998).

24 For a history of ‘metrosexual’ men, see Chapter eight from Paul Deslandes, *The Culture of Male Beauty in Britain: From the First Photographs to David Beckham* (Chicago: University of Chicago Press, 2021).

25 Kenneth Lipartito, ‘Chapter 12: Historical Sources and Data’, in Marcelo Bucheli and R. Daniel Wadhwani, eds, *Organizations in Time: History, Theory, Methods* (Oxford: Oxford University Press, 2013), 289; Adrian Bingham, ‘The Digitization of Newspaper Archives: Opportunities and Challenges for Historians’, *Twentieth Century British History*, 2010, 21, 228; Adrian Bingham, ‘Ignoring the First Draft of History? Searching for the Popular Press in Studies of Twentieth-Century Britain’, *Media History*, 2012, 18, 311–26; Adrian Bingham and Martin Conboy, *Tabloid Century: The Popular Press in Britain, 1896 to the Present* (Pieterlen and Bern: Peter Lang, 2015).

26 ‘The public’, ‘the media’, ‘medical experts’ and ‘consumers’ are not always separate groups of people. As this article will demonstrate, people can fit within all these overlapping and interweaving categories. For a more comprehensive understanding of the ‘public’ in Britain, see Alex Mold *et al.*, *Placing the Public in Public Health in Post-War Britain, 1948-2012* (London: Palgrave Macmillan, 2019); Virgina Berridge, *Marketing Health: Smoking and the Discourse of Public Health in Britain, 1945-2000* (Oxford: Oxford University Press, 2007); Virginia Berridge and Kelly Loughlin, eds, *Medicine, the**Market and**Mass**Media:**Producing**Health in the**Twentieth**Century* (Milton Park: Routledge, 2012); Jane Hand, ‘Marketing Health Education: Advertising Margarine and Visualising Health in Britain from 1964–c.2000’, *Contemporary British History*, 2017, 31, 477–500.

27 Elizabeth Toon, *Private Trauma, Public Drama: Breast Cancer Treatment in Twentieth-Century Britain* (London: Palgrave Macmillan, forthcoming); Hannah J. Elizabeth, ‘“Private Things Affect Other People”: Grange Hill’s Critique of British Sex Education Policy in the Age of AIDS’, *Twentieth Century British History*, 2021, 32, 261–284.

28 Adrian Addison, *Mail Men: The Unauthorized Story of the Daily Mail-The Paper**That Divided and Conquered Britain* (Bloomsbury: Atlantic Books, 2017).

29 Deborah S. Ryan, ‘‘‘All the World and Her Husband”: The *Daily Mail* Ideal home Exhibition 1908-39’, in Maggie Andrews and Mary M. Talbot, eds, *All the World and Her Husband*, *Women In Twentieth-Century Consumer Culture* (London: Cassell, 2000), 12.

30 Shirly Conran, ‘FEMAIL’, *Daily Mail*, 29 October 1968, 15.

31 Adrian Bingham, ‘‘‘The Woman’s Realm”: *The Daily Mail* and Female Readers’, *Daily Mail Historical Archive 1896-2004*, Cengage Learning, 2013.

32 Bingham and Conboy, *Tabloid Century*, 160–62, 195.

33 Alanah Reid, ‘A History of the Daily Mail’, *Historic Newspapers*, 17 September 2020. https://www.historic-newspapers.co.uk/blog/daily-mail-history/ accessed 28 October 2020.

34 Justine Hancock, ‘Femail Body Talk. How to Avoid Those Wedding Belle Blues’, *Daily Mail*, 8 April 1993, 46.

35 William Holmes, ‘Skin-Deep Survey Fails to Convince’, *The Times*, 22 July 1988.

36 Skelly, *The Uses of Excess in Visual**and Material Culture*; Katrina-Louise Moseley, ‘Slimming One’s Way to a Better Self? Weight Loss Clubs and Women in Britain, 1967–1990’, *Twentieth Century British History*, 2020, 31, 427–453.

37 Sheila Jefferys, *Beauty and Misogyny: Harmful Cultural Practices in the West* (Milton Park: Routledge, 2005), 8, 12.

38 From 1980 to 1987, the average quantity of both *BMJ* and *BMJD* sunbed-referencing publications amounted to two per year. In 1988, these two journals published 10 sunbed-related articles—9 were a mixture of research articles, letters and responses from the *BMJ* and 1 was an abstract of the first sunbed-dedicated paper presented at the annual *BMJD* conference. Clearly, sunbed-induced skin cancer concerns had entered both mainstream medical and public spaces; BBC Written Archives Caversham, *Tomorrow’s World*, Series 25, Episode 4, BBC1, 27 October 1988; Dr Ronald Marks, *The Sun and Your Skin* (London: Macdonald & Co, 1988).

39 I. H. Cameron and Christine McGuire, ‘‘‘Are You Dying to Get a Suntan?” – Pre- and Post- Campaign Survey Results’, *Health Education Journal*, 1990, 49, 170.

40 A twentieth-century history of veneral diseases demonstrates similar socio-medical attitudes and responses, Allan M. Brandt, *No Magic Bullet: A Social History of Venereal Disease in the United States since 1880* (Oxford: Oxford University Press, 1987).

41 Clive Seale, ‘Health and Media: An Overview’, *Sociology of Health & Illness*, 2003, 25, 513–31.

42 The history of HIV/AIDs demonstrates that to avoid being stigmatised, some people did not seek medical attention and avoided being tested, Virginia Berridge, *AIDS in the UK: The Making of a Policy, 1981-1994* (Oxford: Oxford University Press, 1996).

43 Graham Stewart, *Bang!:**A**History of Britain in the 1980s* (Bloomsbury: Atlantic Books, 2013), 258, 416–17.

44 Chappell, ‘Loadsamoney Making Fun’, 16.

45 Anita Biressi and Heather Nunn, *Class and Contemporary British Culture* (London: Palgrave Macmillan, 2013), 32–37; Stewart, *Bang!*, 247.

46 Simon Hattenstone, ‘Harry Enfield: “I Don't Like Doing Me”’, *Guardian*, 25 September 2010. https://www.theguardian.com/theguardian/2010/sep/25/harry-enfield-interview accessed 19 December 2019; Anon., ‘Harry Enfield. Loadsamoney. Doin’ up the House’, *Official Charts*. https://www.officialcharts.com/artist/24899/harry-enfield/ accessed 19 December 2019.

47 Henry Taylor, ‘How Old Are You Again? UK Newspaper Age Demographics in 4 Charts’, *The Media Briefing*, 14 August 2014. http://www.themediabriefing.com/article/youth-audiences-newspaper-old-demographics-advertising?curator=MediaREDEF accessed 5 May 2017; Colin Sparks, ‘The Readership of the British Quality Press’, *Media, Culture & Society*, 1987, 9, 432.

48 On Sunday afternoon, the 6 November 1988, *Cigarettes and Chocolate* aired on Radio 4, B. A. Young, ‘Radio: Drama over the Airwaves’, *Financial Times*, 12 November 1988, XXIII.

49 Anthony Minghella, *Minghella Plays: 2: Cigarettes and Chocolate* (New York: Bloomsbury, 1997), 32–33.

50 Sparks, ‘The Readership of the British Quality Press’, 430, 432–33.

51 During the mid-to-late 1980s, Yorkshire Television reportedly served ‘6 million viewers’, including coverage in Scarborough, Hull, Grimsby, Lincoln, Kings Lynn, Ripon, York, Sheffield and Leeds. The TV Room, ‘ITV1 Yorkshire’, 5 August 2011. https://web.archive.org/web/20120219183948/http://www2.tv-ark.org.uk/itvyorkshire/idents.html accessed 11 November 2019.

52 Out of 1,133 votes, the ‘secret meeting’ option won, with 450 votes, *Hollywood Sports*, Yorkshire Television, aired from April 1989 to May 1990; Deborah Ross, ‘The Soap You Can Write as You Watch’, *Daily Mail*, 20 April 1989, 28.

53 Newby Hands, ‘Everything a Judge Should Know about Bimbettes and Their Pouting Older Sisters: The Full Bimbo Teach-In’, *Daily Mail*, 27 January 1990, 12.

54 Mike Williams, ‘“Silicone of Course … and then I Had My Brain ‘Scooped’ and Replaced with Polystyrene Chippings and To Be Honest, Amanda, I Wish I’d had It Done Years Ago”’, *Punch*, 25 May 1990.

55 Anon., ‘Punch’, *New World Encyclopaedia*, 16 June 2019. https://www.newworldencyclopedia.org/entry/Punch_(magazine) accessed 5 December 2019

56 Williams, ‘“Silicone of course”’.

57
*Cinderella Story*, Warner Bros. Pictures, 2004, UK.

58 David Shuttleworth, ‘Sunbeds and the Pursuit of the Year Round Tan: Should Be Discouraged’, *British Medical Journal*, 1993, 307, 1508–09.

59 In the 1990s, confident women in films were rarely depicted as successful ‘career-driven’ women, reaping their own financial reward to pay for their own beauty consumptions. During the early 2000s, on the rare occasions that they were presented, they were presented as either: emotionally cold; undergoing some personal and moral crisis, or they were secretly supported by their wealthy family or a man. Moreover, the American actress Jennifer Coolidge plays a beauty obsessed character in both *Legally Blonde* and *Cinderella Story*; however, in *Legally Blonde* she does not use sunbeds and is depicted as the kind ‘bimbo’ friend, whereas in *Cinderella Story* Coolidge is presented as the ‘evil bimbo’ step-mother who uses sunbeds, *Miss Congeniality*, 2001, UK; *Legally Blonde*, 2001, UK; *Devil Wears Prada*, 2006, UK.

60 David Wilson, *Love and Nausea* (London: Abacus, 1995).

61 B. L. Diffey, ‘Epidemiology and Health Services Research: A Quantitative Estimate of Melanoma Mortality from Ultraviolet a Sunbed Use in the U.K.’, *British Medical Journal of Dermatology*, 2003, 149, 578–81.

62 Berridge, *Demons*; Allan M. Brandt, *The Cigarette Century: The Rise, Fall, and Deadly Persistence of the Product That Defined America* (New York: Basic Books, 2007); Courtwright, *Forces of**Habit*; Tim Madge, *White Mischief: A Cultural History of Cocaine* (Edinburgh and London: Mainstream Publishing, 2001).

63 Shuttleworth, ‘Sunbeds and the Pursuit of the Year Round Tan Should Be Discouraged’.

64 Charles Rosenberg, ‘Illness, Society, and History’, in Charles Rosenberg and Janet Golden, eds, *Framing Disease: Studies in Cultural History: Health and Medicine in American Society* (New Jersey: Rutgers University Press, 1992), xvi, xiii.

65 Helen Keane, *What's**Wrong with**Addiction?* (Melbourne: Melbourne University Press, 2002).

66 Sara Barrett and Jane Kelly, ‘Femail Exclusive. Special report on the Beauty Addicts Whose Desire for a Perfect Body Controls Their Lives. Day One. Women Who Become Hooked on Their Looks’, *Daily Mail*, 27 May 1991, 14–15.

67 Jennifer Crane, *Child Protection in England, 1960–2000:**Expertise*, *Experience, and**Emotion* (London: Palgrave, 2018), 172.

68 Carolyn Jackson and Penny Tinkler, ‘“Ladettes” and “Modern Girls”: “Troublesome” Young Femininities’, *The Sociological Review*, 2007, 55, 251–72; Karen L. Kopelson, ‘Radical Indulgence: Excess, Addiction, and Female Desire’, *Postmodern Culture*, 2006, 17; https://muse.jhu.edu/article/211470/summary; Peder Clark, ‘Claire and Jose Get Off Their Cake: Ecstasy, Raving and Women’s Pleasure in 1990s Britain’, *Cultural and Social History*, 2021. https://www.tandfonline.com/doi/epub/10.1080/14780038.2021.1976702?needAccess=true.

69 Berridge, *Demons*; Courtwright, *Forces of**Habit*.

70 Barrett and Kelly, ‘Special Report on the Beauty Addicts Whose Desire for a Perfect Body Controls Their Lives’, 14–15; Sara Barrett, ‘Femail Exclusive. Concluding Our Special Report on the Beauty Addicts Whose Desire for a Perfect Body Takes Over Their Lives. Confessions of the Cosmetic Junkies’, *Daily Mail*, 28 May 1991, 14–15.

71 Helen McCarthy, *Double Lives: A History of Working Motherhood* (New York: Bloomsbury, 2020).

72 Westcott, ‘I’d Rather Be Wrinkled and Brown than White and Smooth’, 24–25.

73 In 1994 the Department of Health commissioned the omnibus survey of the Office of Population Censuses and Surveys to interview British adults, who were randomly selected and stratified by socioeconomic variables. The survey consisted of 2017 interviews, A. Bulman, ‘Letters. People Are Overusing Sunbeds’, *British**Medical**Journal*, 1995, 310, 1327.

74 Anne Rogers and David Pilgrim, *A Sociology of Mental Health and Illness*, 4th edn (Berkshire: Open University Press, 2010), 75, 77.

75 Keith Wailoo, *How**Cancer**Crossed the**Color**Line* (Oxford: Oxford University Press, 2010).

76 Westcott, ‘I’d Rather Be Wrinkled and Brown than White and Smooth’, 24–25; Liz Frost, *Young**Women and the**Body: A**Feminist**Sociology* (Switzerland: Springer, 2001).

77 Barrett and Kelly, ‘Special Report on the Beauty Addicts Whose Desire for a Perfect Body Controls Their Lives’, 14–15; Jahn L. Forth-Finegan, ‘Sugar and Spice and Everything Nice’, *Journal of Feminist Family Therapy*, 1992, 3, 19–48.

78 Westcott, ‘I’d Rather Be Wrinkled and Brown than White and Smooth’, 24–25.

79 Anon., ‘THE UVABRONZE TAN VERSUS A SPANISH TAN. COULD A LEADING TRAVEL AGENT TELL THE DIFFERENCE?’, *Cosmopolitan*, September 1981, 189.

80 Anon., ‘Femail Bodytalk. The Double Life of the Bit-Part Models’, *Daily Mail*, 14 April 1994, 46–47.

81 Jackie Stacey, *Teratologies*. *Cultural Study of Cancer* (Milton Park: Routledge, 1997), 2, 6, 2; Carsten Timmermann and Elizabath Toon, eds, *Cancer Patients, Cancer Pathways: Historical and Sociological Perspectives* (London: Palgrave Macmillan Ltd, 2012), 62–63, 88.

82 Westcott, ‘I’d Rather Be Wrinkled and Brown than White and Smooth’, 24–25.

83 Anon., ‘Excuses, Excuses … For Using a Sunbed’, *Daily Mail*, 22 January 1994, 3.

84 Elizabeth Haiken*, Venus Envy: A History of Cosmetic Surgery* (Baltimore: John Hopkins University, 1997).

85 Alex Mold, ‘Illicit Drugs and the Rise of Epidemiology during the 1960s’, *Journal of Epidemiology & Community Health*, 2007, 61, 278–81; Virginia Berridge, ‘Passive Smoking and Its Pre-History in Britain: Policy Speaks to Science?’, *Social Science and Medicine*, 1999, 49, 1190, 1188.

86 Catchpole used a sunbed twice a week from only May to December. For three or four years, Brimmell had used a sunbed for 4 h every week. Wieck used a sunbed ‘at least once a week’. For 7 years, Sayles had used a sunbed once or twice a week, Westcott, ‘I’d Rather Be Wrinkled and Brown than White and Smooth’, 24–25.

87 Wieck asserted that she used sunbeds ‘in moderation’ and ‘always looked carefully at the dosage time’. To tan ‘safely’, she read the sunbed literature and always wore goggles and protective cream. Sayles also used protective gel and cream before and after her sunbed session, Westcott, ‘I’d Rather Be Wrinkled and Brown than White and Smooth’, 24–25.

88 Jan Shure, ‘Choosing a Health Club’, *Daily Mail*, 14 October 1980, 12.

89 Barrett, ‘Concluding Our Special Report on the Beauty Addicts Whose Desire for a Perfect Body Takes Over Their Lives’, 14–15.

90 Hancock, ‘Femail Body Talk. How to Avoid Those Wedding Belle Blues’, 46.

91 Anon., ‘The Double Life of the Bit-Part Models’, 46–47; Brandt and Rozin, *Morality and Health*.

92 Westcott, ‘I’d Rather Be Wrinkled and Brown than White and Smooth’, 24–25.

93 Richard Duce, ‘Sunbed Users Ignore Danger’, *The Times*, 9 March 1994, 7.

94 David Goodhart, ‘Sunbed Caused Skin Cancer’, *Financial Times*, 8 March 1994, 13; Jenny Hope, ‘A Woman’s Skin Cancer Is Blamed on Sunbed’, *Daily Mail*, 8 March 1994, 6; Louise Atkinson, ‘A Healthy, All Year Tan – or Basking in Ignorance about the Risk of Skin Cancer’, *Daily Mail*, 9 March 1994, 17.

95 Dr Farr’s statement is weakened when correlated with a review of severe sunbed incidents from the early-to-mid 1980s as only affluent consumers could afford household sunbeds during that period. In 1985, for instance, the annual report of the Department of Trade and Industry's Home Accident Surveillance System stated that, after stairs, the main cause of incidents at home was attributed to sunbeds. During that year alone, a total of 143 sunbed incidents were reported, Kenneth Gosling, ‘Peril of Summer Weekends / Accidents in the Home’, *The Times*, 5 November 1986, 3.

96 Courtwright, *Forces of**Habit.*

97 Hansen, ‘Shades of Change’, 192.

98 Jessica Mosebach, ‘A Content Analysis of Gender Differences in Newspaper Book Reviews’ (unpublished PhD thesis, University of Nevada, 2007), 20.

99 Anon., ‘Sunbed Launch Off after Cancer Scare’, *Daily Mail*, 10 March 1994, 21.

100 Anon., ‘Watching Brief’, *Guardian*, 15 April 1994, 52; On the BBC News, two dermatologists, Dr Peter Farr and Dr Clifford Lawrence, used the Newcastle incident to confirm that sunbeds caused skin cancer, BBC Broadcast Archive, ‘NEWS – SUNBED CAUSES SKIN CANCER’, BBC News, Aired 6 p.m., 7 March 1994.

101 In 1996 alone, another two ‘tanorexic’ examples emerged in the *Daily Mail*. Both women were 27 years old and working-class (a widowed shop assistant and a maritally ‘single’ sales executive), Tooze, ‘Tanorexia’, 44–45; Eleanor Bailey, ‘Burning Ambitions’, *Guardian*, 27 January 1995, 9; Dan Glaister, ‘Tanorexics’, *Guardian*, 12 April 1996, 15; Duce, ‘Sunbed Users Ignore Danger’, 7; *3-D, BURNING ISSUE - HEALTHY CHOICE*?, ITV Yorkshire Television, 8 May 1997; *Beauty Consumer Watchdog*, BBC1, 8 January 1996; *Esther*, ‘Tanorexia’, BBC 2, 18 June 1997; *The Vanessa Show*, ‘Addicted to Sunbeds’, ITV, 13 October 1996.

102 Between 2000 and 2010, this article was well cited by medical researchers in several different journals. These included *Psychology & Marketing*, *Cancer Causes & Control*, the *BMJD*, *Photodermatology, Photoimmunology & Photomedicine*, *Health Education Research* and *Health Promotion International*. The researchers did not critique Fiala’s findings; instead they cited the article to justify that women, more so than men, used sunbeds to achieve their aesthetic goals, as they perceived a tan as ‘healthy’ and ‘attractive’. One dermatologist even reiterated that young women tanned their bodies ‘possibly to protect themselves from the fear of close relationships’, Fiala, Kopp and Gunther, ‘Why Do Young Women Use Sunbeds?, 950; Brian Diffey, ‘Sunbeds, Beauty and Melanoma’, *British Medical Journal of Dermatology*, 2007, 157, 215.

103 Liz Gill, ‘My Warning to Anyone Who Is Tempted to Use a Sunlamp’, *Daily Mail*, 1 April 1997, 40.

104 Westminster Hall Debates, ‘Sunbed Use: Health Implications — [Stewart Hosie in the Chair]’, 13 February 2019. https://www.theyworkforyou.com/whall/?id=2019-02-13b.341.0 accessed 28 October 2020.

105 Fiala, Kopp and Gunther, ‘Why Do Young Women Use Sunbeds?’; Heckman and Manne, *Shedding**Light on Indoor Tanning*, 58; Mary Baker *et al.*, ‘The Effect of Initial Indoor Tanning With Mother on Current Tanning Patterns’, *Archives of Dermatology*, 2010, 146, 1427–28.

106 Ian Murray (Medical Correspondent), ‘Sunbed Clients may be Paying with Their Lives’, *The Times*, 3 March 1999, 6; The sample base of this study consisted of 6,143 adults. Taylor Nelson, ‘Consumer Research on Sun tanning and Sunbeds’, The Sunbed Association, UK, 1997.

107 For instance, the Bronz factory in London’s West End deliberately provided ‘discreet surroundings’ for men who used sunbeds, Wellcome Trust Collection, Box 628, Sunbathing ephemera Box 1, The Bronz Factory leaflet, London, 27 June 1992.

108 Henrike E. Karim-Kos *et al.*, ‘Recent Trends of Cancer in Europe: A Combined Approach of Incidence, Survival and Mortality for 17 Cancer Sites since the 1990s’, *European Journal of Cancer*, 2008, 44, 1365.

109 Dummer *et al., Skin Cancer*, 14–15; National Cancer Intelligence Network and Cancer Research UK, ‘Cancer by Deprivation in England 1996 – 2011’, 3 June 2014. http://www.ncin.org.uk/about_ncin/cancer_by_deprivation_in_england accessed March 2020; Homung and Poorsattar, ‘Tanning Addiction’.

## Notes

Fabiola Creed is a health historian specialising in the history of twentieth-century Britain, tanning technologies, public health, stigma and mass media. This article is based on her PhD research, which explored the history of the sunbed industry. She is currently a Research Fellow on Professor Hilary Marland’s project ‘The Last Taboo of Motherhood?: Postnatal Mental Disorders in Twentieth-Century Britain’ at Warwick (funded by Wellcome Trust).

